# Fungal Infections in Renal Transplant Patients

**DOI:** 10.14740/jocmr2104w

**Published:** 2015-04-08

**Authors:** Asif Khan, Elie El-Charabaty, Suzanne El-Sayegh

**Affiliations:** a Department of Medicine, Staten Island University Hospital, 475 Seaview Ave., Staten Island, NY 10305, USA; b Department of Nephrology, Staten Island University Hospital, 475 Seaview Ave., Staten Island, NY 10305, USA

**Keywords:** Renal transplant, Fungal infection, Transplant

## Abstract

Organ transplantation has always been considered to be the standard therapeutic interventions in patients with end-stage organ failure. In 2008, more than 29,000 organ transplants were performed in US. Survival rates among transplant recipients have greatly improved due to better understanding of transplant biology and more effective immunosuppressive agents. After transplant, the extent of the immune response is influenced by the amount of interleukin 2 (IL-2) being produced by the T-helper cells. Transplant immunosuppressive therapy primarily targets T cell-mediated graft rejection. Calcineurin inhibitor, which includes cyclosporine, pimecrolimus and tacrolimus, impairs calcineurin-induced up-regulation of IL-2 expression, resulting in increased susceptibility to invasive fungal diseases. This immunosuppressive state allows infectious complication, leading to a high mortality rate. Currently, overall mortality due to invasive fungal infections (IFIs) in solid organ transplant recipients ranges between 25% and 80%. The risk of IFI following renal transplant is associated with the dosage of immunosuppressive agents given, environmental factors and post-transplant duration. Most fungal infections occur in the first 6 months after transplant because of the use of numerous immunosuppressors. *Candida* spp. and *Cryptococcus* spp. are the yeasts most frequently isolated, while most frequent filamentous fungi (molds) isolated are *Aspergillus* spp. The symptoms of systemic fungal infections are non-specific and early detection of fungal infections and proper therapy are important in improving survival and reducing mortality. This article will provide an insight on the risk factors and clinical presentation, compare variation in treatment of IFIs in renal transplant patients, and evaluate the role of prophylactic therapy in this group of patients. We also report the course and management of two renal transplant recipients admitted to Staten Island University Hospital, both of whom developed pulmonary complications secondary to Aspergillus infection.

## Introduction

Renal transplantation has always been considered to be the standard therapeutic interventions in patients with end-stage organ failure. In 2008, more than 29,000 organ transplants were performed in US [1]. Increased immunosuppression, multiple organ transplantation as well as environmental factors are just some of the risk factors involved in this increased incidence of invasive fungal infection (IFI) [2]. Survival rates among transplant recipients have greatly improved due to better understanding of transplant biology and more effective immunosuppressive agents.

The Transplant-Associated Infection Surveillance Network conducted a 5-year prospective study among 1,063 organ transplant recipients. One thousand two hundred eight were diagnosed with IFI. The most common IFIs were invasive candidiasis (53%), invasive aspergillosis (IA) (19%), cryptococcosis (8%), non-Aspergillus molds (8%), endemic fungi (5%), and zygomycosis (2%) [3].

With the use of more effective immunosuppressors, most fungal infection occurs within the first 6 months after transplant [4]. The median time to the onset of infection is also dependent on the causative agents. The median times to the onset of candidiasis, aspergillosis, and cryptococcosis after transplant were 103, 184, and 575 days respectively [3].

In the US, each year approximately 15,000 renal transplants are performed, with a 5-year survival rate of > 80% [5]. The rate of infection in renal transplant patients during the initial 3 years after transplant is 46% and is associated with the duration and net dosage of immunosuppressive agents given [1].

Five percent of all infections in renal transplant recipients are fungal in origin [6]. Fungal conidia are being constantly inhaled eliminated by innate mechanisms in healthy individuals. Aspergillus spores are ubiquitous in the environment and may become concentrated in hospital ventilation systems. Aspergillus was regarded as a weak pathogen, but there has been a dramatic increase in the incidence of aspergillosis in the last two decades, yet mortality has decreased from 92% in 1990 to 60% in 1998 [7].

IA is a life-threatening complication in patients who undergo solid organ transplantation, having an incidence between 0.5% and 2.2% [8] with a mortality rate of > 70% [9], and a high case-fatality rate of up to 88% [10].

## Presentations

Renal transplant recipients usually present with vague clinical symptoms of infection and early laboratory reveals are normal. To improve the prognosis, a high index of suspicion is necessary in renal transplant recipients.

Unexplained fever despite broad-spectrum antibiotic treatment for more than 3 - 6 days, recurring febrile episodes after initial defervescence, or the presence of pulmonary infiltrates during antibiotic treatment can indicate a fungal infection. Early symptoms of invasive pulmonary aspergillosis include cough, fever and hemoptysis.

Preventive measures should be taken using sensitive assays (e.g. antigen detection and molecular assays) to monitor patients at given interval and stop progression to invasive disease. A positive assay will require initiation of therapy and reduction in the anti-suppression medication, with frequent monitoring of the patient.

To reduce the risk of infection, pre-transplant screening of donor and recipient, vaccination, and post-transplant surveillance and prophylaxis are required.

## Risk Factors in Renal Transplant Recipients

Aspergillus spores are ubiquitous in the environment. Hospital constructions or at adjacent sites predispose the hospital ventilation systems to become concentrated with Aspergillus spores and may serve as the source micro-epidemics of aspergillosis.

In a retrospective case-control study on 156 transplant cases, early-onset IA (i.e., occurred during the first 90 days after transplantation) was identified in 57% cases and 43% cases had late-onset infections (i.e., occurred after 90 days period) [11]. This bimodal pattern of infection is suggestive of different risk factors between early- and late-onset cases.

The use of vascular amines for > 24 h after surgery, intensive care unit (ICU) readmission, development of renal failure, need for hemodialysis, and occurrence of > 1 episode of bacterial infection after transplantation were the major risk factors for early-onset IA [11].

Late-onset IA was contributed to age > 50 years, chronic impaired graft function, use of immunosuppressive drugs and occurrence of an immunosuppression-related neoplasm [11]. Other risk factors such as diabetes and prolonged pre-transplant dialysis had also shown to promote serious fungal infections [12].

## Diagnosis

The European Organization for Research and Treatment of Cancer/Invasive Fungal Infections Cooperative Group and the National Institute of Allergy and Infectious Diseases Mycoses Study Group (EORTC/MSG) Consensus Group defines invasive fungal infection when histological analysis or culture of a specimen of tissue taken from a site of disease can reveal fungus. In case of *Cryptococcus neoformans*, a positive result of an India ink preparation of CSF or detection of capsular antigen in CSF is sufficient [13].

Diagnosis of fungal infection in transplant recipients can be very perplexing due to the vague patient symptoms and the lack of specificity of blood test or radiologic finding. It is estimated about 30% of the IFI cases at death remain undiagnosed or untreated [14]. An elevated creatinine level in RTR indicates a state of chronic rejection and opportunistic infection [6]. A combination of various methods and regular screening is therefore advisable to ensure that a diagnosis is reached as soon as possible.

Tests including cultures from blood, cerebrospinal fluid, peritoneal fluid, quantitative tests (such as sandwich enzyme-linked immunosorbent assays or molecular assays) that directly detect the protein products or nucleic acids of the organisms are needed for routine monitoring of transplant patients [15].

The galactomannan assay can detect aspergillosis before symptoms appear, but sensitivity and specificity in solid organ transplant patients are lower than in hematological patients [16]. A negative result does not rule out the diagnosis of IA and repeat testing is recommended.

Having a baseline serum tested and testing biweekly for increasing galactomannan antigen levels can monitor therapeutic response. False negative results can occur due to decline in antigen levels in response to antimicrobial therapy [14].

In addition to routine component of initial evaluation, invasive procedures that provide tissues for culture and histologic testing should be performed early.

Potassium hydroxide wet mount smear is the most sensitive screening test for the rapid detection of fungal elements. All tissues from patients with a suspected invasive fungal infection should be stained with a fungal stain such as acridine orange, periodic acid-Schiff reaction, Grocott-Gomori methenamine silver staining, lectins, and Calcofluor white [15].

Imaging studies are not always useful in helping clinicians diagnose fungal infections. Thoracic computed tomographic scans, chest radiographs, and abdominal ultrasonography can be used to reveal pulmonary infiltrates, hydronephrosis, fungus balls, or perinephric abscesses highly suggestive of transplant failures from infections.

The results of those tests would enable clinicians to individualize prophylactic antifungal regimens and minimize drug-associated toxicity.

## Antifungal Treatment in Renal Transplant Recipients

One of the most important goals in the field of transplantation is the prevention of fungal infections. The epidemiological exposure and immunosuppression state of the patient should be determined for individual risk assessment, thereby allowing for the implementation of preventive interventions, taking account of the potential side effects and emerging resistance of the antifungal prophylactic strategies. Table 1 displays the drugs frequently used to treat renal mycoses [15].

**Table 1:** Drugs Frequently Used to Treat Renal Mycoses Are Listed Below [15]

Type of infection	Drug	Dosage	Duration of treatment
Candiduria	Fluconazole	200 - 400 mg/day	Several days before and after the procedure
Candidemia	Fluconazole	Loading dose: 800 mg/day, then 400 mg/day	14 days after first negative blood culture result
Invasive aspergillosis	Voriconazole	4 mg/kg twice daily	Until all signs and symptoms of infection have resolved for at least 2 weeks
Cryptococcosis	Fluconazole	400 mg/day	6 - 12 months
Mucormycosis	Liposomal amphoteracin B	5 mg/kg/day	Until patient exhibits a favorable response

### Amphotericin B

Amphotericin B deoxycholate (AmB-D) is a polyene with a very broad spectrum of activity, including most yeasts and filamentous fungi. For the past four decades, AmB-D has been considered to be the standard antifungal agent for treatment of IA in severely immunocompromised patients. But more than 50% of cases have reported nephrotoxicity or infusion-related side effects. AmB-D is not recommended as a first-line therapy in renal transplant recipients [17].

In a separate study of 239 immunosuppressed AmB-treated patients (63% of whom had undergone a transplant procedure), S-Cr level doubled from baseline in 53% of patients and increased to > 2.5 mg/dL in 29% of patients, whereas 15% of patients required dialysis [18]. The risk of nephrotoxicity increases with the concurrent use of calcineurin. Other side effects include fevers, chills, nausea and vomiting, and hypotension.

### Liposomal amphotericin B (L-AmB)

L-AmB is a lipid-associated formulation of the broad-spectrum polyene antifungal agent amphotericin B. L-AmB is indicated for the treatment of severe systemic mycoses in which nephrotoxicity limits the use of amphotericin B.

It is active against clinically relevant yeasts and molds, including *Candida* spp., *Aspergillus* spp. and filamentous molds such as zygomycetes. Liposomal formulation is also indicated for the empirical treatment of presumed fungal infections, in whom the fever has failed to respond to broad-spectrum antibiotics and appropriate investigations have failed to define a bacterial or viral cause.

A meta-analysis study reported reduction in all-cause mortality for invasive fungal infections by almost 30% by lipid-associated formulations when compared with AmB-D [19].

In a separate study by Leenders et al demonstrated higher efficacy of L-AmB given at 5 mg/kg than AmB-D in a study of patients with IA [20].

### Azole

All azole agents interrupt the cell membrane ergosterol synthesis by fungi by inhibition of cytochrome P450. Elevations in hepatic enzyme levels alanine aminotransferase and aspartate aminotransferase occur with azole therapy. Although most patients have asymptomatic elevation of hepatic enzyme levels, liver function tests prior to therapy, within the first 2 weeks after the initiation of therapy, and then every 2 - 4 weeks throughout therapy should be performed [21].

### Voriconazole

This triazole antifungal agent, with broadened antifungal spectrum, is used for opportunistic infections in immunocompromised patients. Voriconazole shows good *in vitro* activity against all Candida species, including certain Candida strains that are inherently fluconazole-resistant, and strains of *Candida albicans* that have acquired resistance to fluconazole and other yeasts, including *Cryptococcus neoformans* [21].

Voriconazole should not replace fluconazole or other antifungal agents for treatment of most Candida infections. The drug has more side effects and drug interactions than fluconazole.

It has also shown activity against Aspergillus, including amphotericin B-resistant Aspergillus strains. A large, randomized trial compared standard amphotericin B with voriconazole for primary treatment of IA [22]. Of 277 patients who had confirmed IA and who received ≥ 1 dose of study drug, 133 were randomized to receive amphotericin B and 144 were randomized to receive voriconazole.

Successful outcomes were noted in 32% of patients in the amphotericin B group versus 53% of patients in the voriconazole group (95% CI: 10.4 - 32.9). The survival rate was 58% in the amphotericin B group when compared to 71% in the voriconazole group (P = 0.02). Based on the results voriconazole was found to be more effective than amphotericin B and likely to become the drug of choice for the primary treatment of patients with IA.

It is available in both oral and intravenous formulations and the recommended regimen loading dose of 6 mg/kg every 12 h for two doses, followed by a maintenance dose of 4 mg/kg every 12 h. Metabolism of voriconazole occurs via CYP 450 enzyme in the liver and therefore it is necessary to adjust dose in patients with hepatic insufficiency [21].

Reversible “disturbance of vision” is the most common side effect of voriconazole and occurs in 30% of patients. Fortunately these symptoms tend to decrease or disappear in spite of continued therapy in most patients [21].

In a randomized, double blind, placebo-controlled, crossover study in kidney transplant recipients with stable renal function, co-administration of voriconazole in patient receiving cyclosporine resulted in increased minimum plasma concentration of cyclosporine by 1.7 folds [23]. The sample size in the study was not significant to draw a conclusion.

Therefore it is recommended to decrease the cyclosporine dose by half when voriconazole is initiated in patients. Blood cyclosporine concentrations should also be monitored and increased if voriconazole is discontinued. Of interest, patients are advised to avoid fatty meals as they decrease the bioavailability of voriconazole.

The major overall advantage of the azoles over amphotericin B is their lower toxicity and availability for oral administration. The co-administration of voriconazole with calcineurin inhibitors is an alternative, although dose reduction of immunosuppressive drugs and close monitoring of drug levels is required [4].

### Fluconazole

Fluconazole is a triazole antifungal drug used in the treatment and prevention of superficial and systemic fungal infections. Fluconazole is available in both oral and intravenous formulations. It is the most common antifungal prophylactic agent.

However empirical treatment with fluconazole in neutropenic patients with suspected fungal infection may be inappropriate, because prior exposure, as treatment or prophylaxis, is associated with resistant candidal strains. In addition, fluconazole has limited activity against IA [24].

### Itraconazole

Itraconazole has a wider spectrum than fluconazole. It is active against both yeasts and molds. Boogaerts et al compared itraconazole with AmB-D in the empirical treatment of neutropenic sepsis. Itraconazole arm had fewer adverse events (5% versus 54%), and less withdrawal because of adverse events (19% versus 38%) and nephrotoxicity (5% versus 24%) when compared with AmB-D arm [25].

Side effects included headache, dizziness, raised hepatic transaminases, gastrointestinal symptoms, peripheral neuropathy and allergic reactions.

### Posaconazole

Posaconazole is the newest triazole antifungal agent, approved by FDA as prophylaxis for invasive Aspergillus and Candida infections in patients aged ≥ 13 years [26]. It is only found in oral formulation and predominantly eliminated in the feces, so dose adjustment is not required in renal and hepatic insufficiency. Posaconazole inhibits hepatic cytochrome P 450-3A4; therefore dose adjustments must be made to immunosuppressive drugs [26].

The investigation involving 602 neutropenic patients with acute myelogenous leukemia or myelodysplastic syndrome undergoing myelosuppressive chemotherapy, compared 29 days of prophylactic posaconazole with other azoles. Posaconazole was more effective in prevention of IA, when compared to other azoles (P < 0.001) [26].

### Candins

The candins are a new class of antifungal agent that disrupts the biosynthesis glycan polymers in fungal cell wall. This specificity for glycan linkages makes it less toxic to human cells with a high therapeutic index for this class of compounds. With antifungal activity against Candida and Aspergillus species and *Pneumocystis carinii*, these drugs have the potential use both in the treatment and in the prophylaxis of invasive fungal infections in solid organ transplant recipients [27].

### Micafungin

Using a prospective, randomized, double blind comparative trial with 882 subjects, van Burik compared micafungin to fluconazole for prophylaxis against IFI. The overall success rate was 80% for micafungin when compared to 73.5% for fluconazole (95% CI: 0.9 - 12; P = 0.03). Moreover, treatment duration was shorter for micafungin and was effective in reducing the need for empirical treatment [27]. In conclusion, micafungin is as effective as fluconazole and poses as an alternative for antifungal prophylaxis.

### Caspofungin

Caspofungin is licensed for the treatment of IA in adult patients who are refractory to or intolerant of AmB-D, lipid formulations of amphotericin B and/or itraconazole. A favorable response was noted in 45% of 83 patients [17]. Caspofungin is also licensed for empirical therapy for presumed fungal infections (such as Candida or Aspergillus) in febrile neutropenic adults.

A comparison study was done between caspofungin and L-AmB in a randomized controlled trial involving over 1,000 neutropenic patients. Caspofungin was found to be as effective, but generally better tolerated than L-AmB [28].

### Anidulafungin

Anidulafungin is not metabolized by or eliminated through the liver or kidney. Consequently anidulafungin is free of interactions with other drugs such as prednisone, cyclosporin, tacrolimus, mofetil or sirolimus that are metabolized through the liver. Equally, dosage adjustments are not required in renal impaired patients and in patients with severe liver disease. When compared to caspofungin, anidulafungin has a wider spectrum of action and lower toxicity profile. Although experience is still limited, and more studies need to be conducted, anidulafungin will be highly useful in the clinical management of solid organ transplant recipients.

### Nystatin

Nystatin is a polyene antifungal medication that is active against molds and yeast infections, most notably Candida. To determine potential use of nystatin as a prophylactic or therapeutic drug in immunocompromised patients, 12 randomized trials were conducted comparing nystatin to placebo, untreated control group and fluconazole and amphotericin.

It was found that nystatin was similar to placebo in terms of potency (relative risk 0.85, 95% CI: 0.65 - 1.13) and inferior to fluconazole in preventing invasive fungal infection (relative risk 0.37, 0.15 - 0.91). Therefore, nystatin cannot be recommended for prophylaxis or treatment of IFI [29].

### Flucytosine

Flucytosine is used in the treatment of systemic fungal infections caused by sensitive organisms mainly Candida and Cryptococcus. Flucytosine is known for rapid emergence of resistance when used alone; therefore it is given in combination with another antifungal agent.

Side effects include nausea, diarrhea, hepatotoxicity and bone marrow suppression. All are reversible on discontinuation of the drug.

## Human Interferon (IFN)-γ Therapy

Armstrong-James’s study involving 10 renal allograft recipients with proven or probable invasive fungal diseases was studied over a 5-year period [29]. As all renal transplant patients were taking immunosuppressive therapy, the IFN-γ levels were much lower in stable transplants than in healthy controls (P < 0.001).

But comparing stable renal allograft recipients with renal allograft recipients who had developed an invasive fungal disease revealed patients with invasive fungal diseases failed to mount an adequate IFN-γ response to the fungal infection [30].

Based on the findings, it may be concluded that T cell-based immune responses are important for protective immunity against invasive fungal diseases in transplant patients.

Additionally, a separate study was conducted by the same author, looking at the potential benefits of adding adjunctive human IFN-γ therapy to on seven renal transplant patients who developed life-threatening, disseminated IFIs refractory to conventional antifungal drug therapy [31].

Six out of seven patients had a promising response to 6 weeks of human IFN-γ therapy. Furthermore, long-term patient follow-up reported no relapse history. Currently prolong antifungal therapy in IFI patient can be very expensive. In contrast, the 6-week course of IFN-γ injections will be less expensive with an approximate cost of $2,600 [31].

Therefore, these observations provide a possible explanation for the therapeutic benefit of adjunctive human IFN-γ therapy in renal allograft recipients with invasive fungal diseases. Till date, research and clinical studies with the implications of adjunctive human IFN-γ therapy are still limited, and more studies need to be conducted.

## Granulocyte-Colony Stimulating Factor (G-CSF)

The use of G-CSF and granulocyte monocyte-colony stimulating factor (GM-CSF) can shorten the period of neutropenia, and improve the overall immune status of the patient.

## Antifungal Prophylaxis

Study was conducted by Playford to determine the effects of antifungal prophylaxis in solid organ transplant recipients. Fourteen randomized controlled trials were studied with 1,497 randomized subjects [32].

Antifungal prophylaxis failed to show a reduction in mortality (RR 0.90, 95% CI: 0.57 - 1.44). However, a significant reduction in IFIs was demonstrated in liver transplant recipients using fluconazole (RR 0.28, 95% CI: 0.13 - 0.57). A conclusion in renal transplant recipients could not be drawn due to the insufficient data [32].

One should take into account the high mortality rates of IFI in transplant recipients, or situations where the individual risk is great, antifungal prophylaxis should be considered empirically. The duration of prophylaxis is currently unknown, but it has been recommended that this should cover the period of neutropenia [22]. An approach to antifungal prophylaxis for organ transplant recipients is shown in Table 2 [33].

**Table 2:** Suggested Approach to Antifungal Prophylaxis for Organ Transplant Recipients [33]

Transplant organ	Targeted pathogen	High-risk characteristics	Agent	Duration
Liver	Aspergillus	Poor allograft function; fulminant hepatic failure pretransplantation; reexploration or retransplantation; hemodialysis; isolation of aspergillus from any site	Lipid AmB	1 - 4 weeks
Liver	Candida	Repeated operation; higher intraoperative transfusion requirements; longer operation time; renal failure; ICU stay	Fluconazole alternative: echinocandin or lipid AmB	1 - 4 weeks
Lung	Aspergillus	Airway specimen cultures positive for aspergillus, particularly for patients with rejection or poor graft function; increased immunosuppression	Itraconazole or voriconazole or lipid-AmB A (full dose I/V) ± nebulized AmB	Depends on CT findings and clearance of sputum cultures, appearance of tracheal anastomosis. 1 to 6 months
Pancreas	Candida	All procedures (risk increased with enteric drainage, anastomotic leak, pancreas transplantation after kidney transplantation, pancreatitis)	Fluconazole alternative: echinocandin or lipid AmB	4 weeks
Bowel	Candida	All procedures (risk increased with peritonitis or leaks, reexploration, renal failure, ischemia, CMV infection, parenteral nutrition)	Fluconazole alternative: echinocandin or lipid AmB	2 - 4 weeks

Voriconazole is approved by the FDA for the treatment of aspergillosis. This azole offers broader filamentous mold activity than either fluconazole or itraconazole but has no activity against the agents of mucormycosis. In addition, voriconazole is a significant inhibitor of the cytochrome P450 enzymes.

The use of fluconazole prophylaxis (400 mg/day) has been shown to reduce the incidence and severity of Candida infections [34]. Fluconazole does not have activity against filamentous fungi. Drug interactions with calcineurin inhibitor are variable; dose readjustment is essential to prevent graft rejection.

Itraconazole (2.5 mg/kg twice daily) has been compared to fluconazole in this group of patients and found to be comparable in preventing Candida infection but more effective in preventing mold infection [35].

Amphotericin B (both regular and lipid formulations) can be used for the prevention of invasive fungal infections. However, low dose amphoteracin B regimens as a prophylaxis for IA have been futile [36].

### Intravascular lines

Any intravascular cannulae may act as the port of entry for fungal infections. A reduction of overall mortality from candidemia following cannula removal was 40%, compared with 86.9% when cannulae were not removed. Intravascular cannulae removal therefore is recommended in the treatment of candidemias whenever possible [37].

### Vaccines

Due to the immunocompromised state of transplant recipients, they do not respond to vaccines when compared to healthy individuals. Therefore all transplant candidates must have their vaccine status assessed and updated as per the standard recommendations by Centers for Disease Control and Prevention. Patients who are not immune to varicella should also be vaccinated, with a minimum of 2 - 4 weeks series completion before transplantation [1].

### Environmental

In areas with high incidence of infection due to aspergillosis or Candida species, both fungal prophylaxis and epidemiological protection (e.g. HEPA filtered air supply in hospital) may be utilized.

## Cases

### Case 1

A 58-year-old female with an extensive past medical history of multiple comorbidities with renal transplant done 5 years ago, taking cellcept (myophenolate), prograft (tacrolimus) and prednisolone was admitted with progressive worsening dyspnea associated with non-resolving cough and low grade fever. She had a bronchoscopy done 2 months back that revealed aspergillosis fumigatus.

On physical examination, she had a temperate of 100.8 °F with bilateral rhonchi and coarse breath sounds. She was immediately admitted to the ICU, and intubated for airway protection until antibiotics and antifungal agents took effect.

Her initial set of lab showed elevated WBC count of 41.45 and a Cr of 1.14. Aspergillus Ab was negative. Blood and urine culture was negative. Urine RME revealed few yeast.

A chest X-ray was done and reported left upper lobe opacity.

A CT chest showed multiple focal air space opacity (Lt > Rt) and small bilateral pleural effusion.

Patient was started on levofloxacin, amikacin, meropenem, and voriconalzole among her usual medications. Her prograft dose was reduced from 2 mg to 1 mg.

Bronchoscopy was done 9 days after admission. Broncho-alveolar lavage from the left lower lobe did not identify any fungus and fungal Ag markers were negative. Right thoracoscopy wedge biopsy was done, but all tests were normal.

The patient responded well to the treatment and was successfully extubated. The WBC count came down to normal levels. She was able to ambulate without the need for oxygen. She was discharged with cellcept and prograft and she was asked to continue voriconalzole for 3 weeks more.

### Case 2

A 56-year-old female from a nursing home, with multiple comorbidities with a significant past medical history of renal transplant 2 months back and taking cellcept (myophenolate), prograft (tacrolimus) and prednisolone, comes to the ER with the shortness of breath and cough associated with green sputum for 1 day and rectal bleeding.

On examination, the patient was moderately distressed and cachectic. She was hypotensive (85/50 mm Hg) with a pulse of 92 bpm. The patient was immediately resuscitated, appropriate labs were ordered and patient was started on empirical antibiotics, vancomycin and cefepime.

On admission, CBC was significant for neutropenia with a total WBC count of 2.17, Hb of 8.2 and Hct of 27.2. A cell count 2 days prior to her admission was normal. Her fall in counts may be due to sepsis or side effect of cellcept.

Chest X-ray showed right lower lobe consolidation. Chest CT reported bilateral basilar and lower lobe consolidation and small right apical pneumonia. CT of abdomen was suggestive of sigmoid colitis.

Echo showed possible tricuspid vegetation, which could be a source for the patient’s septicemia.

Bronchoscopy was done the next day. Diffuse inflammation was noted on the tracheobronchial tree and bronchoalveolar lavage testing was positive for clusters of septate fungal organisms suggestive of aspergillosis; Candida was also identified. BAL culture grew *Pseudomonas aeruginosa*.

A repeat CBC on the second day of admission revealed pancytopenia. Patient was treated with valgancyclovir, vancomycin, meropenem and neupogen. Her cellcept was put on hold, until the cell count stabilizes. She was transferred to Mount Sinai after 2 days from admission, where she was diagnosed with aspergillosis, and was treated accordingly.
